# Screening of antimicrobial, antioxidant properties and bioactive compounds of some edible mushrooms cultivated in Bangladesh

**DOI:** 10.1186/s12941-015-0067-3

**Published:** 2015-02-07

**Authors:** Mohammed Mehadi Hassan Chowdhury, Khadizatul Kubra, Sheikh Rashel Ahmed

**Affiliations:** Department of Microbiology, Noakhali Science and Technology University, Sonapur, Noakhali, 3814 Bangladesh; Department of Biotechnology and Genetic Engineering Sonapur, Noakhali Science and Technology University, Sonapur, Noakhali, Bangladesh; Department of Genetic Engineering and Biotechnology, University of Chittagong, Chittagong, Bangladesh

## Abstract

**Background:**

For a long time mushrooms have been playing an important role in several aspects of the human activity. Recently edible mushrooms are used extensively in cooking and make part of new food in Bangladesh for their beneficial properties. The aim of this study is to screen some values of mushrooms used in Bangladesh.

**Methods:**

Methanolic extracts of 3 edible mushrooms (*Pleurotus ostreatus, Lentinula edodes*, *Hypsizigus tessulatus)* isolated from Chittagong, Bangladesh were used in this study. Phenolic compounds in the mushroom methanolic extracts were estimated by a colorimetric assay. The antioxidant activity was determined by radical 1, 1-diphenyl;-2-picrylhydrazyl (DPPH) radical scavenging assay. Eight microbial isolates were used for antimicrobial activity of methanolic extract of mushrooms by the agar well diffusion method with slight modification.

**Results:**

Determination of antimicrobial activity indicated considerable activity against all bacteria and fungi reveling zone of inhibition ranged from 7 ± 0.2 to 20 ± 0.1 mm. Minimum inhibitory concentration values of the extracts showed that they are also active even in least concentrations ranged from 1 mg/ml to 9 mg/ml. *Lentinula edodes* showed the best antimicrobial activity than others. *Pseudomonas aeruginosa* was quite resistant and *Saccharomyces cerevisiae* was more sensitive than others microbial isolates. Antioxidant efficiency by inhibitory concentration on 1,1-Diphenly-2-picrylhydrazyl (DPPH) was found significant when compared to standard antioxidant like ascorbic Acid . The concentration (IC50) ranged from 100 ± 1.20 to 110 ± 1.24 μg/ml. Total phenols are the major bioactive component found in extracts of isolates expressed as mg of GAE per gram of fruit body, which ranged from 3.20 ± 0.05 to 10.66 ± 0.52 mg/ml. Average concentration of flavonoid ranged from 2.50 ± 0.008 mg/ml to 4.76 ± 0.11 mg/ml; followed by very small concentration of ascorbic acid (range, 0.06 ± 0.00 mg/ml to 0.21 ± 0.01 mg/ml) in all the isolates. All the isolates showed high phenol and flavonoid content (except *Pleurotus ostreatus*), but ascorbic acid content was found in traces.

**Conclusion:**

This study has revealed that selected edible mushrooms have not only nutritional values but also some therapeutic values. Proper and more investigations can lead us to use these as strong medicine in future.

## Introduction

Mushrooms have become attractive as a functional food and as a source for the development of drugs and nutraceuticals [1]. Edible mushrooms are the fleshy and edible fruit bodies of several species of macrofungi. Edibility may be defined by criteria that include absence of poisonous effects on humans and desirable taste and aroma [2,3]. Edible mushrooms are consumed by humans as comestibles for their nutritional value and they are occasionally consumed for their supposed medicinal value. Mushrooms consumed by those practicing folk medicine are known as medicinal mushrooms [4].

Medicinal mushrooms are thought to be treatments for diseases, yet remain unconfirmed in mainstream science and medicine, and so are not approved as drugs or medical treatments [5].

Preliminary research has shown some medicinal mushroom isolates to have cardiovascular, anticancer, antiviral, antibacterial, antiparasitic, anti-inflammatory, and antidiabetic properties [6,7]. Currently, several extracts have widespread use in Japan, Korea and China, as potential adjuvants to radiation treatments and chemotherapy [5,8]. Major medicinal properties attributed to mushrooms include anticancer, antibiotic, antiviral activities, immune response stimulating effects and blood lipid lowering effects [9]. Both fruiting body and mycelium of different mushrooms contain different compounds such as terpenoids, steroids, polyphenol, polyketides, polyglucan, flavonoids, alkaloids, polysaccharides and dietary fibers which exert several pharmacological activities [10]. They are the rich source of antioxidants, antibiotics and antineoplastic activity [11].

The history of mushroom cultivation is not so long in Bangladesh; however, mushroom consumption is increasing rapidly in this country due to cheap source of dietary protein, carbohydrate, vitamins and minerals. For the medicinal and nutritional value, several species of mushroom are being cultivated in Bangladesh [12]. These are reishi, enoki, oyster, shiitake, shimeji, milky, beech and nameko etc. [13].

*Hypsizygus tessulatus* which is commonly known as Shimeji mushroom contains glucans, niacin, vitamin B and D. Vitamin B aids in immunity and weight loss, while vitamin D is deemed to be effective against osteoporosis and anti-aging [14]. The previous name of this mushroom was *Hypsizygus marmoreus* and it also contain different biologically active compounds like polysaccharides, l-ergothioneine, sterols, ergosterol - provitamin D_2_ which have different medicinal uses [15-17].

*Pleurotus ostreatus*, the oyster mushroom, is a common edible mushroom. The oyster mushroom may be considered a medicinal mushroom, since it contains statins such as lovastatin which work to reduce cholesterol [18].

*Lentinula edodes* known as Shiitake is an edible mushroom native to East Asia, which is cultivated and consumed in many Asian countries. It is considered a medicinal mushroom in some forms of traditional medicine [19]. Studies there indicate prolonged survival and improved quality of life when gastric cancer patients with unrespectable or recurrent diseases are treated with lentinan in combination with other chemotherapies [20].

The aim of this study was to evaluate the antibacterial, cytotoxic and antioxidant properties of *Hypsizygus tessulatus, Lentinula edodes* and *Pleurotus ostreatus* different extract both from submerged culture and mycelia mat.

## Materials and method

### Mushroom samples

Samples of *P. ostreatus, Hypsizygus tessulatus, Lentinula edodes* were collected which were cultivated and sold as edible mushrooms from Chittagong, Bangladesh. Identification was done by comparing their morphological, anatomical and physiological characteristics and monographs with descriptions given in the manual [21]. Then mushrooms were air-dried in a liophylizator before analysis. All the specimens were deposited at the herbarium of mycology laboratory, Department of Microbiology, University of Chittagong, Chittagong, Bangladesh. The fruiting bodies, carefully removed from the hyphae were weighed then dried at 40°C for 24 h. The dried fruiting body samples were weighed and ground into powder prior to extraction.

### Preparation of methanolic extracts

Preparation of methanolic extracts of mushrooms was done based on procedures described by Barros *et al.* [22] with some modifications. A fine-dried mushroom powder sample (100 g) was extracted by stirring with 100 ml of methanol at 25°C at 150 rpm for 24 hours and filtered through Whatman no. 4 paper. The residue was then extracted with two additional 100-ml portions of methanol. The combined methanolic extracts were evaporated at 40°C to dryness. The organic solvent in the extracts was removed by a rotary evaporator. For the entire analysis, compounds of extract were dissolved in dimethylsulfoxide (DMSO), and filter-sterilization was done through a 0.22-μm membrane filter. Extracts were kept in the dark at 4°C for not more than 1 week prior to use.

### Determination of bioactive components

Phenolic compounds in the mushroom methanolic extracts were measured by a colorimetric assay, wherein 1 ml of sample was mixed with 1 ml of Folin and Ciocalteu’s phenol reagent. After 3 minutes, 1 ml of saturated sodium carbonate solution was added to the mixture and adjusted to 10 ml with distilled water [22]. The reaction was kept in the dark for 90 minutes, after which the absorbance was measured spectrophotometrically at 765 nm. Gallic acid was used to calculate the standard curve (0.01-0.4 mM). The mean values of results were expressed as milligrams of gallic acid equivalents (GAEs) per gram of extract.

Flavonoid concentrations were estimated [23], where in methanolic extract solution (1 ml) was diluted with 4.3 ml of 80% aqueous ethanol, and 0.1 ml of 10% aluminum nitrate and 0.1 ml of 1 M aqueous potassium acetate were added. After 40 minutes at room temperature, the solution was mixed well, and the intensity of the pink color was measured spectrophotometrically (Model - UV-1601 SHIMADZU) at 415 nm. Quercetin was used to calculate the standard curve of total flavonoid concentration absorbance = 0.002108 μg quercetin – 0.01089 (R2: 0.9999).

Ascorbic acid was determined [22], where in methanolic extract (100 mg) was extracted with 10 ml of 1% metaphosphoric acid for 45 minutes at room temperature and filtered through Whatman no, 4 filter paper. The filtrate (1 ml) was mixed with 9 ml of 2, 6-dichloroindophenol (0.025%), and the absorbance was measured within 30 minutes at 515 nm against a blank. Content of ascorbic acid was calculated on the basis of the calibration curve of authentic L-ascorbic acid (0.020-0.12 mg/mL). The results were expressed as milligrams of ascorbic acid per milliliter of extract. All the above estimations of bioactive compounds were carried out in triplicate and means were plotted.

### Scavenging activity on DPPH for antioxidant assay

The antioxidant activity of the extracts on the stable radical 1, 1-diphenyl;-2-picrylhydrazyl (DPPH) was determined by the method developed by Feresin *et al*. (2002) [24]. The 0.1 ml of mushroom ethanol extract, at various concentrations was added to 3 ml of a 0.004% methanol solution of DPPH and was allowed to stand for 30 min for the reaction to occur. The absorbance of the resulting solution was measured at 517 nm from this values the corresponding percentage of inhibitions were calculated by using the following equation:

$$ \mathrm{I}\ \%=\left[\left({\mathrm{A}}_{\mathrm{Blank}}-{\mathrm{A}}_{\mathrm{sample}}\right)/{\mathrm{A}}_{\mathrm{Blank}}\right]\times 100, $$

Where, A_blank_ is the absorbance of the control reaction (containing all reagents except the test sample) and A_sample_ is the absorbance of sample/standard.

Extract concentration providing 50% inhibition (IC_50_) was calculated from the graph plotted I% versus concentration curve. The free radical scavenging activity was measured for different concentrations of sample and compared with standard (Ascorbic acid).

### Determination of antimicrobial efficacy

#### Microbial test organisms

The bacterial test organisms used were *Staphylococcus aureus* (ATCC 25923), *Bacillus subtilis* (ATCC 6633) as gram-positive species and *Escherichia coli* (ATCC 25922), *Klebsiella pneumonia* (ATCC 13883), *Pseudomonas aeruginosa* (ATCC 27853) and *Salmonella typhi* (ATCC 33459) as gram-negative species grown in Mueller Hinton agar at 37°C for 24 hours (Merck). Fungal species was unicellular *Candida albicans* (ATCC 60192) and *Saccharomyces cerevisiae* (ATCC 9763) grown in potato dextrose agar (Hi Media) at ambient temperature for 72 hours. The bacterial isolates were multidrug resistant determined by disc diffusion assay according to Iwalokun et al. [25].

#### Antibacterial and antifungal screening

Antimicrobial activity of methanolic extract of mushrooms was determined by the agar well diffusion method [26], with slight modification to suit the conditions of this experiment. Briefly, the methanol extracts were dissolved in 3% dimethylsulfoxide (DMSO) to a final concentration of 10 mg/ml, and filter sterilized through 0.45-μm membrane filter. Small wells (6 mm in diameter) were made in the agar plates by sterile cork borer. One hundred microliters of the extract of each isolate of mushrooms was loaded into the different wells. An overnight culture of each microbial isolate was emulsified with nutrient broth to a turbidity that was equivalent to 0.5 McFarland (108 cfu/ml). In order to determine the antimicrobial efficacy of the fractions, aliquot of test culture (100 μl) was evenly spread over the surface of the solidified agar. Bacteria were cultured on Mueller Hinton agar; and fungi on potato dextrose agar. Ciprofloxacin (5 μg/well) for bacteria, fluconazole (5 μg/well) for fungi were used as positive control and DMSO was used as negative control for test microorganisms. All the preloaded plates with respective extract and test organism were incubated at 37°C, for 24 hours for bacteria and at 27°C. 48 hours for fungi. After incubation period, zone of inhibition was measured in millimeters. All the tests were carried out in triplicate and their means recorded.

### Minimum inhibitory concentration (MIC)

The standard agar dilution protocol with doubling dilution was used. The extract was incorporated into nutrient agar at concentrations ranging from 0.39 mg/ml to 25 mg/ml. A control without the extract was also prepared. 10 μL of each test organisms, previously diluted to 10 CFU/ml, were used to inoculate the plates. These were incubated at 37°C for 24 h in the first instance and for another 24 h before the growth was observed and recorded. The minimum inhibitory concentrations (MICs) of the extract for each test microorganism were considered the agar plate with the lowest concentrations without growth [27].

## Results

### Antimicrobial

The present study has revealed the antibacterial and antifungal activity of mushroom extract. The results of antibacterial activity were presented in Table 1. All the mushrooms also revealed antimicrobial activity showing different MICs for each microorganism (Table 2).

**Table 1:** Preliminary antimicrobial testing of mushroom extracts through determination of zone of inhibition (mm ± SD)*

**Isolates**	**Mushroom extracts**			**Standard drug**
	***Pleurotus ostreatus***	***Lentinula edodes***	***Hypsizigus tessulatus***	
*Staphylococcus aureus*	13.5 ± 0.1	16 ± 0.2	14 ± 0.2	25 ± 0.2
*Bacillus subtilis*	14 ± 0.2	17 ± 0.2	16 ± 0.2	24 ± .01
*Escherichia coli*	11 ± 0	14 ± 0.1	12.5 ± 0.1	24 ± .01
*Pseudomonas aeruginosa*	7 ± 0.2	12 ± 0.2	9 ± 0.2	20 ± 0.1
*Salmonella typhi*	11.5 ± 0.3	15 ± 0.1	12.5 ± 0.1	20 ± 0.1
*Klebsiella pneumoniae*	9.7 ± .02	13 ± 0.1	11.5 ± 0.3	24 ± 0.1
*Candida albicans*	13.5 ± 0.1	18 ± 0.2	16 ± 0.2	25 ± 0.3
*Saccharomyces cerevisiae*	15 ± 0.2	20 ± 0.1	18 ± 0.2	30 ± 0.3

*The diameters of zone of inhibition were expressed in millimeter (mm) as mean ± standard deviation (SD).

**Table 2:** **Minimum inhibitory concentrations determinations of extracts**

**Isolates**	**MIC (mg/ml)**		
	***Pleurotus ostreatus***	***Lentinula edodes***	***Hypsizigus tessulatus***
*Staphylococcus aureus*	6	3	7
*Bacillus subtilis*	7	3	8
*Escherichia coli*	6	3	7
*Pseudomonas aeruginosa*	8	4	9
*Salmonella typhi*	5	2	7
*Klebsiella pneumoniae*	7	3	6
*Candida albicans*	4	1	6
*Saccharomyces cerevisiae*	4	1	5

Note. Each value is expressed as mean (*n* = 3) and standard deviations were less than 5%.

### Antioxidental

In case of antioxidant screening of ethanol extract of *Hypsizygus tessulatus* powdered mycelium has shown in Table 3. The IC_50_ value, defined as the concentration of antioxidant required for 50% scavenging of DPPH radicals in this specified time period, is a parameter widely used to measure antioxidant activity; a smaller IC_50_ value corresponds to a higher antioxidant activity of the plant extract. IC_50_ value of the ethanol extract was 105.0 μg ml^−1^. These values state that *H. tessulatus* has moderate DPPH scavenging activity compared to Ascorbic acid standard.

**Table 3:** **Free radical scavenging activity (IC**_**50**_ **μg/ml)**

**Sample**	**Value**
*H. tessulatus* Extract	105.0 ± 1.23
*Pleurotus ostreatus* Extract	100 ± 1.20
*Lentinula edodes* Extract	110 ± 1.24
Standard Ascorbic Acid	5.25 ± 0.21

The values of IC_50_ are expressed as Mean ± SEM (n = 3).

### Bioactive compounds

Table 4 showed phenol, flavonoid and ascorbic acid concentrations of the isolates; total phenols were the major bioactive component found in extracts of isolates expressed as mg of GAE per gram of fruit body, which ranged from 10.66 ± 0.52 to 5.65 ± 0.05 mg/ml.

**Table 4:** **Bioactive compound contents of the wild edible mushrooms (Mean ± Standard Deviation; n = 3)**

**Mushroom sample**	**Total Phenols(mg/ml)**	**Total Flavonoids (mg/ml)**	**Ascorbic acid (mg/ml)**
*H. tessulatus*	5.65 ± 0.05	2.50 ± 0.008	0.11 ± .01
*Pleurotus ostreatus*	3.20 ± 0.05	00	0.06 ± 0.00
*Lentinula edodes*	10.66 ± 0.52	4.76 ± 0.11	0.21 ± 0.01

## Discussion

### Antimicrobial assay

All the mushrooms used in this study were found to exhibit various degrees of antimicrobial effects against the tested microorganisms. The zone of inhibition exhibited more than 10 millimeters was considered as highly active for extracts. *P. ostreatus* has a broad-spectrum antibacterial and antifungal activity. Similar antimicrobial potentials have been observed in the culture extracts of *Irpex lacteus* (Rosa *et al*., 2003), *Agrocybe* sp. (Kavanagh *et al*., 1950; Mavoungou *et al*., 1987) [28-30]. The best *in-vitro* antibacterial activity was by *L. edodes* (17 ± 0.2mm) followed by *Pleurotus ostreatus and Hypsizigus tessulatus* against *Bacillus subtilis. Lentinula edodes* also showed best antimicrobial activity than *Pleurotus ostreatus and Hypsizigus tessulatus.* This antimicrobial efficacy of *Lentinula edodes* was similar of the study of Kuznetsov *et al*., 2005 [31].

Ishikawa *et al*. (2001), reported data similar to the results obtained in this work, showing antibacterial action of *L. edodes* against *B. cereus*, *S. aureus* and *E.coli* [32]. Komemushi *et al*. (1996) also reported that *L. edodes* inhibited growth of Gram positive and Gram negative bacteria [33]. The revealed information regarding strong antimicrobial activity of *Hypsizigus tessulatus* against pathogenic isolates has similarity of the study of Hearst *et al*., 2009 [34].

*Candida albicans* and *Saccharomyces cerevisiae* was found to be very susceptible when compared to positive controls. *Pseudomonas aeruginosa* showed more resistant to mushrooms extract comparatively than other isolates.

Among the bacterial isolates, The MIC of all mushrooms extract was high for *Pseudomonas aeruginosa.* The MICs of *Lentinula edodes* against all isolates were low comparatively others. The Lowest MIC was observed for *Candida albicans and Saccharomyces cerevisiae* against *Lentinula edodes.* This quite similar result was also observed by Barros *et al.*, 2006 [35].

### Antioxidantal

In the DPPH assay, the antioxidants are able to reduce the stable DPPH radical (purple) to the non-radical form DPPH-H (yellow). The DPPH scavenging activities of antioxidants are attributed to their hydrogen donating abilities. All the mushroom extract showed the antioxidant activity. But the differentiation between the extract is too close. *Lentinula edodes* showed the highest antioxidant activity. This result is quite similar by Emanuel Vamanu, 2012 [36].

The result for antioxidant activity of *P. ostreatus* is also quite similar by Arbaayah HH And Umi Kalsom Y, 2013 [37].

### Bioactive components

Flavonoids, which are also phenolic compounds, were not detected in *P. ostreatus.* Our observation agrees with findings of Mattila *et al.*, 2001 [38] and according to USDA, mushrooms are regarded as non-sources of flavonoids. Higher contents of bioactive compounds were found in *Lentinula edodes*. This statement is too similar to the experiment by Calhelha *et al.*, 2007 [22]. The amount of phenolic contents and Flavonoids from *H. tessulatus* was moderate and similar by the study of Monira *et al.*, 2012 [39].

## Conclusion

The present investigation can conclude that the methanolic extract of 3 edible mushrooms showed biopharmaceutical potentiality. However whether such extracts will act as effective therapeutic agents remain to be investigated, the identification of the bioactive compounds and study of mechanisms of actions are necessary prior to application.

## References

[1] Barros L, Ferreira MJ, Queiros B, Ferreira ICFR, Baptista P (2007). Total phenols, ascorbic acid, β-carotene and lycopene in Portuguese wild edible mushrooms and their antioxidant activities. Food Chem.

[2] Arnora D. Mushrooms demystified 1986. Ten Speed Press. p. 23. ISBN 0-89815-169-4

[3] Mattila P, Suonpää K, Piironen V (2000). Functional properties of edible mushrooms. Nutrition.

[4] Ejelonu OC, Akinmoladun AC, Elekofehinti OO, Olaleye MT (2013). Antioxidant profile of four selected wild edible mushrooms in Nigeria. J Chem Pharm Res.

[5] Sullivan R, Smith JE, Rowan NJ (2006). Medicinal mushrooms and cancer therapy: translating a traditional practice into Western medicine. Perspect Biol Med.

[6] Benedict RG, Brady LR (1972). Antimicrobial activity of mushroom metabolites. J Pharm Sci.

[7] Iwalokun BA, Usen UA, Otunba AA, Olukoya DK (2007). Comparative phytochemical evaluation, antimicrobial and antioxidant properties of *Pleurotus ostreatus*. Afr J Biotech.

[8] Borchers AT, Krishnamurthy A, Keen CL, Meyers FJ, Gershwin ME (2008). The immunobiology of mushrooms. Exp Biol Med.

[9] Bobek P, Galbavy S (2008). Effect of pleuran (β-glucan from *Pleurotus ostreatus*) on the antioxidant status of the organism and on dimethylhydrazine-induced precancerous lesions in rat colon. Br J Biomed Sci.

[10] Breene WM (1990). Nutritional and medical value of specialty mushrooms. J Food Protec.

[11] Akyuz M, Kirbag S (2009). Antimicrobial activity of *Pleurotus eryngii* var. ferulae grown on various agro-wastes. EurAsia J BioSci.

[12] Amin SMR, Alam N (2008). Study of mycelial growth of *Cordyceps sinensis* in different media, at different PH level and temperature. Bangladesh J Mushroom.

[13] Imtiaj A, Rahman SA (2008). Economic viability of mushrooms cultivation to poverty reduction in Bangladesh. Trop Subtrop Agroecosyst.

[14] Ikekawa T, Saitoh H, Feng W, Zhang H, Li L, Matsuzawa T (1992). Antitumour activity of *Hypsizygus marmoreus*. 1. Antitumour activity of extracts and polysaccharides. Chem Pharm Bull.

[15] Tsuchida K, Aoyagi Y, Odani S, Mita T, Isemura M (1995). Isolation of a novel collagen-binding protein from the mushroom, *Hypsizigus marmoreus*, which inhibits the lewis lung carcinoma cell adhesion to type IV collagen. J Biol Chem.

[16] Saitoh H, Feng W, Matsuzawa T, Ikekawa T (1997). Antitumour activity of *Hypsizygus marmoreus*. 11. Preventive effect against lung metastasis of Lewin lung carcinoma. Yakugaku Zasshi.

[17] Matsuzawa T, Saitoh H, Sano M, Tomita I, Ohkawa M, Ikekawa T (1998). Studies on antioxidant effects of *Hypsizigus marmoreus*. II. Effects of *Hypsizigus marmoreus* for antioxidant activities of tumor-bearing mice. Yakugaku Zasshi.

[18] Gunde-Cimerman N, Cimerman A (1995). Pleurotus fruiting bodies contain the inhibitor of 3-hydroxy-3-methylglutaryl-coenzyme A reductase-lovastatin. Exp Mycol.

[19] Borchers AT, Stern JS, Hackman RM, Keen CL, Gershwin ME (1999). Mushrooms, tumors, and immunity. Proc Soc Exp Biol Med.

[20] Ina K, Kataoka T, Ando T (2013). The use of lentinan for treating gastric cancer. Anti Cancer Agents Med Chem.

[21] Purkyastha RP, Aindrila C (1978). Manual of Indian edible mushrooms.

[22] Calhelha RC, Vaz JA, Ferreira ICFR, Baptista P, Estevinho LM (2007). Antimicrobial activity and bioactive compounds of Portuguese wild edible mushrooms methanolic extracts. Euro Food Res Technol.

[23] Opige M, Kateyo E, Kabasa JD, Olila D (2006). Antibacterial activity of extracts of selected indigenous edible and medical mushrooms of eastern Uganda. Int J Trop Med.

[24] Feresin GE, Tapia A, Angel GR, Delporte C, Erazo NB, Schmeda-Hirschmann G (2002). Free radical scavengers, anti-inflammatory and analgesic activity of *Acaena magellanica*. J Pharm Pharmacol.

[25] Iwalokun BA, Ogunledun A, Ogbolu DO, Bamiro SB, Jimi-Omojola J (2004). *In vitro* antimicrobial properties of aqueous garlic extract against multidrug-resistant bacteria and *Candida* species from Nigeria. J Med Food.

[26] Oyetayo VO, Dong CH, Yao YJ (2009). Antioxidant and antimicrobial properties of aqueous extract from *Dictyophora indusiata*. Open Myco J.

[27] Oboh IE, Akerele JO, Obasuyi O (2007). Antimicrobial activity of the ethanol extract of the aerial parts of *Sida acuta* burm.f. (malvaceae). Trop J Pharm Res.

[28] Rosa LH, Machado KMG, Jacob CC, Capelkari M, Rosa CA, Zani CL (2003). Screening of Brazillian basidiomycetes for antimicrobial activity. Mem Inst Oswaldo Cruz.

[29] Kavanagh F, Hervey A, Robbins WJ (1950). Antibiotic substances from basidiomycetes. VI Agrocybe dura Proc Natl Acad Sci USA.

[30] Mavoungou H, Porte M, Oddoux L (1987). Active antitumoraled des myceliums d’Agrocybe dura, Mycoacia uda et Phanerochaete laevis. Ann Pharmaceutic Francaises.

[31] Kuznetsov OIU, Mil’kova EV, Sosnina AE, Sotnikova NIU (2005). Antimicrobial action of *Lentinus edodes* juice on human microflora *Zh* Mikrobiol. Epidemiol Immunobiol.

[32] Ishikawa NK, Kasuya MCM, Vanetti MCD (2001). Antibacterial activity of *Lentinula edodes* grown in liquid medium. Braz J Microbiol.

[33] Komemushi S, Yamamoto Y, Fujita T (1996). Purification and identification of antimicrobial substances produced by *Lentinus edodes*. J Antibacterial Antifungal Agents.

[34] Hearst R, Nelson D, McCollum G, Millar BC, Maeda Y (2009). An examination of antibacterial and antifungal properties of constituents of Shiitake (*Lentinula edodes*) and oyster (*Pleurotus ostreatus*) mushrooms. Complement Ther Clin Pract.

[35] Barros L, Calhelha RC, Vaz JA, Ferreira ICFR, Baptista P, Estevinho LM (2006). Antimicrobial activity and bioactive compounds of Portuguese wild edible mushrooms methanolic extracts. Eur Food Res Technol.

[36] Vamanu E (2012). In vitro antimicrobial and antioxidant activities of ethanolic extract of lyophilized mycelium of *Pleurotus ostreatus* PQMZ91109. Molecules.

[37] Arbaayah HH, Umi Kalsom Y (2013). Antioxidant properties in the oyster mushrooms (*Pleurotus* spp.) and split gill mushroom (*Schizophyllum commune*) ethanolic extracts. Mycosphere.

[38] Mattila P, Konko K, Eurola M, Pihlava JM, Astola J, Vahteristo L (2001). Contents of vitamins, mineral elements, and some phenolic compounds in cultivated mushrooms. J Agric Food Chem.

[39] Monira S, Haque A, Muhit A, Sarker NC, Alam AHMK, Rahman AA (2012). Antimicrobial, antioxidant and cytotoxic properties of *Hypsizygus tessulatus* cultivated in Bangladesh. Res J Med Plant.

